# Targeting E3 ubiquitin ligases and their adaptors as a therapeutic strategy for metabolic diseases

**DOI:** 10.1038/s12276-023-01087-w

**Published:** 2023-10-02

**Authors:** Yelin Jeong, Ah-Reum Oh, Young Hoon Jung, HyunJoon Gi, Young Un Kim, KyeongJin Kim

**Affiliations:** 1 Department of Biomedical Sciences, College of Medicine, Inha University, Incheon, Republic of Korea; 2 Program in Biomedical Science & Engineering, Inha University, Incheon, Republic of Korea; 3 Research Center for Controlling Intercellular Communication (RCIC), College of Medicine, Inha University, Incheon, 22212 Republic of Korea

**Keywords:** Mechanisms of disease, Metabolic disorders

## Abstract

Posttranslational modification of proteins via ubiquitination determines their activation, translocation, dysregulation, or degradation. This process targets a large number of cellular proteins, affecting all biological pathways involved in the cell cycle, development, growth, and differentiation. Thus, aberrant regulation of ubiquitination is likely associated with several diseases, including various types of metabolic diseases. Among the ubiquitin enzymes, E3 ubiquitin ligases are regarded as the most influential ubiquitin enzymes due to their ability to selectively bind and recruit target substrates for ubiquitination. Continued research on the regulatory mechanisms of E3 ligases and their adaptors in metabolic diseases will further stimulate the discovery of new targets and accelerate the development of therapeutic options for metabolic diseases. In this review, based on recent discoveries, we summarize new insights into the roles of E3 ubiquitin ligases and their adaptors in the pathogenesis of metabolic diseases by highlighting recent evidence obtained in both human and animal model studies.

## E3 Ligases: A New Frontier in Metabolic Disease Therapy

This review highlights the crucial role of E3 ubiquitin ligases and their adaptors in regulating metabolic diseases, such as obesity, diabetes, and nonalcoholic fatty liver disease. The authors discuss the mechanisms controlling E3 ubiquitin ligases and their adaptors, as well as the potential of targeting specific E3 ubiquitin ligases or adaptors for therapeutic interventions. By developing therapeutic approaches that target these proteins, it may be possible to stabilize target proteins without affecting the expression of others, resulting in more effective treatments for metabolic diseases with fewer adverse effects. Further understanding of the complex biochemistry underlying the physiological functions regulated by E3 ubiquitin ligases and their adaptors is necessary to identify their potential roles as metabolic regulators and therapeutic targets.

## Introduction

Metabolic diseases are heterogeneous diseases characterized by diverse dysregulated biological processes, including increased adiposity, insulin resistance, diabetes, hyperinsulinemia, hypertension, nonalcoholic fatty liver diseases (NAFLD), and dyslipidemia, which are related to accelerated atherosclerosis and cardiovascular disease (CVD)^1^. Although the underlying mechanisms of their pathogenesis are not fully understood, uncontrolled amounts of proteins, fat, and other substances play a crucial role in accelerating metabolic diseases^2^. Therefore, the regulation of specific protein levels could be an attractive option to control the pathogenesis of metabolic diseases. This can be achieved by regulating the expression or activity of proteins involved in the various cellular processes that are dysregulated due to aberrant posttranslational modifications, which are associated with the manifestation of metabolic diseases.

**Fig. 1:**
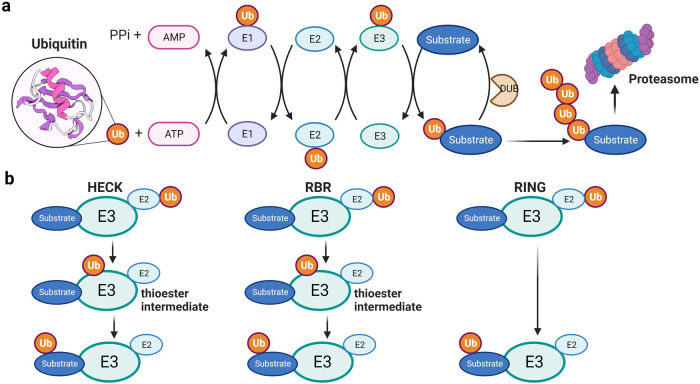
The ubiquitin proteasome system and classification of E3 ubiquitin ligases. **a** Ubiquitination of proteins is achieved through an enzymatic cascade involving ubiquitin-activating enzyme (E1), ubiquitin-conjugating enzyme (E2) and ubiquitin ligase (E3). E3 ubiquitin ligases provide platforms for binding E3 enzymes and specific protein substrates, thereby coordinating ubiquitination of the target protein. **b** The HECT and RBR types transfer ubiquitin to its active site cysteine to form a thioester intermediate before transferring ubiquitin to the substrate protein. The RING type transfers ubiquitin directly from E2 to the substrate protein.

The ubiquitin‒proteasome system (UPS) is a key mechanism for the spatiotemporal control of metabolic enzymes or dedicated regulatory proteins^3^. UPS-mediated degradation, which irreversibly eliminates enzymes, is especially important for cells to acclimate to changes in their internal or external environment. The process of ubiquitin-mediated proteasomal degradation involves various components, including the 76-amino acid protein ubiquitin (Ub), Ub-activating enzyme (E1), Ub-conjugating enzyme (E2), ubiquitin ligase (E3), deubiquitinating enzyme (DUB) and proteasome^4^ (Fig. 1a). Given that E3 ubiquitin ligases are crucial in determining the specific target proteins that undergo degradation via ubiquitination, their activity, expression, and turnover are tightly regulated to prevent inappropriate ubiquitination and cellular dysfunction^5^. Emerging evidence suggests that the activity and functional regulation of E3 ubiquitin ligases are controlled by accessory proteins or adaptor proteins^6^, highlighting the importance of E3 ubiquitin ligases and their adaptors in the development of metabolic diseases. This review will classify and discuss the underlying mechanisms of action of E3 ubiquitin ligases and their adaptors in a broad range of metabolic diseases.

## Classificiation of E3 ubiquitin ligases

Over 600 E3 ubiquitin ligases have been identified thus far, and each targets specific substrate proteins involved in various cellular processes, such as cell death, DNA repair, tumorigenesis, immunity, and metabolism^7^. Dysregulated functions of E3 ubiquitin ligases have been linked to many human diseases, making them potential targets for drug development. Based on the characteristic domain and mechanism of ubiquitin transfer to substrate proteins, all E3 ubiquitin ligases can be classified into three main families: the homologous to E6AP C-terminus (HECT) family, the really interesting new gene (RING) between RING (RBR) family, and the RING finger family (Fig. 1b).

### HECT family E3 ubiquitin ligases

The HECT family of E3 ubiquitin ligases contains a HECT catalytic domain with an N-terminal lobe (N-lobe) that contains the E2 binding domain and a C-terminal lobe (C-lobe) carrying the catalytic cysteine. The C-lobe can move around via a flexible hinge region to facilitate Ub transfer from E2 to E3^8^. Based on the domain organization in the N-terminal region, there are three main subfamilies of HECT E3 ubiquitin ligases. The NEDD4 family is the best characterized, consisting of nine human members that share a similar domain structure with a membrane/lipid-binding C2 domain, two to four WW domains for substrate recognition, and a C-terminal HECT domain^9^. The second class is identified by one or more regulators of chromatin condensation 1 (RCC1)-like domain (RLD), which serve as a guanine nucleotide exchange factor (GEF) for the small GTPase in membrane trafficking processes^10^. This family consists of six members that can be subdivided into four “small” and two “large” HERCs, where HERC1 and HERC2 are the largest HECT E3 ubiquitin ligases with approximately 5000 residues. The remaining 13 HECTs are classified as other HECT ligases and do not share specific domains at the N-terminus^10,11^.

Several adaptor proteins have emerged as potent regulators of HECT E3 ubiquitin ligases, as they can influence E3 localization and catalytic activity and regulate E3-substrate interactions^12^. For example, NEDD4 family members are found in different subcellular localizations and have several substrates lacking the classic proline-rich PY motif for interaction with the WW domains of NEDD4 protein^13^. These adaptors are critical because many contain PY motifs that facilitate efficient substrate targeting by the E3 ubiquitin ligase. Additionally, several other adaptors utilize different binding domains on E3 ubiquitin ligases, affecting phosphorylation or ubiquitination, and alter E2-E3 interactions to regulate ligase activity. Increasing evidence shows that adaptors play prominent roles in disease-relevant processes, including various cancers, neurological disorders, immunological diseases, and metabolic diseases^14^.

### RBR family E3 ubiquitin ligases

The RBR ubiquitin ligases are the smallest E3 ubiquitin ligase family, consisting of only 14 members. They possess three domains: a RING1 domain that binds to Ub-loaded E2, a RING2 domain that catalyzes a transthioesterification reaction with a cysteine residue and receives ubiquitin from RING1, and an in-between-RING (IBR) domain^15^. The process of ubiquitination by RBR E3 ubiquitin ligases occurs sequentially. First, RING1 recognizes the E2-Ub conjugate and transfers the Ub to the catalytic cysteine in RING2 to create a thioester intermediate, which is then transferred to the substrate^16^. In recent years, several subfamilies of RBR E3 ubiquitin ligases have been identified, including the Ariadne family, which includes Ariadne homolog 1 (ARIH1), ARIH2, PARkin-like cytoplasmic protein (PARC), and ankyrin repeat and IBR domain-containing protein 1 (Ankib1) and has the Ariadne domain. Other members, such as Parkin, RANBR2-type and C3HC4-type zinc finger-containing 1 (RBCK1) and HOIL-1 interacting protein (HOIP), possess various other domains^17^. Ankib1 has an RBR domain located near the center of its structure, while the RBR domain is located at the N-terminus of the RNF19A and RNF19B proteins.

The human genome encodes approximately 14 members of the RBR E3 ubiquitin ligase family, including ARIH1, ARIH2, Ankib1, PARC, Parkin protein 2 (PARK2), RBCK1, RNF14, RNA19A, RNF19B, RNF31, RNF144A, RNF144B, RNF216, and RNF217^18^. Dysfunction of RBR E3 ubiquitin ligases has been implicated in various diseases, including inflammation, neurodegenerative diseases, cancer, and metabolic disorders^19^.

### RING finger family E3 ubiquitin ligases

**Fig. 2:**
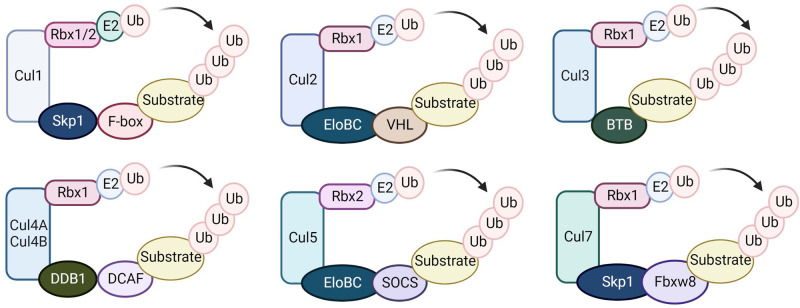
The Cullin-RING E3 ligase family. Cullin proteins recruit adaptor proteins (Skp1 for Cul1 and Cul7, Elongin B/C for Cul2 and Cul5, BTB protein for Cul3, and DDB1 for Cul4A and 4B), receptor proteins (F-box proteins for Cul1, VHL-box for Cul2, DCAF for Cul4A and 4B, SOCS for Cul5, and FBXW8 for Cul7), and the RING protein (Rbx1/2) to form CRL E3 ubiquitin ligases that transfer ubiquitin from Rbx1/2-bound E2 to substrate proteins.

RING finger E3 ubiquitin ligases are the largest E3 family and are characterized by the RING or U-box catalytic domain, which directly transfers ubiquitin from E2 to a substrate protein^20^. The cullin-ring ubiquitin ligase (CRL) family is the largest subfamily of RING E3 ubiquitin ligases, with over 200 members, and is responsible for approximately 20% of all ubiquitination in cells^21^. CRLs use cullin proteins as a central scaffold, which binds to the RING-box protein and an adaptor protein-substrate receptor complex through its C- and N-terminal domains, respectively^22^. CRL1, also known as SCF (Skp1-Cul1-F-box) complexes, uses ~70 F-box proteins as substrate recruiters^23^. Each CRL subfamily has a distinct set of adaptors or substrate recruiters. While Cul1 interacts with Skp1 to recruit substrates, Cul2 and Cul5 utilize VHL (von Hippel‒Lindau)- and SOCS (suppressor of cytokine signaling)-box proteins, respectively, as substrate adaptors via the adaptor complex Elongin B/C^24^. CRL3 uses BTB/POZ (broad complex, tramtrack and bric-à-brac/poxvirus and zinc finger)-domain proteins as both adaptor and substrate receptors, which recognize substrates using their meprin and TRAF homology (MATH) motif or Kelch beta-propeller repeat^25^. Cul4A and Cul4B use DNA damage-binding protein 1 (DDB1) as an adaptor and Ddb1- and Cul4-associated factors (DCAFs) as substrate recruitment subunits^26,27^. Cul7 and Cul9 are the most recently identified members of the cullin family and bind to Skp1 and Fbxw8^22,28^. Cul7, similar to Cul1, utilizes F-box proteins as substrate recruiters, while Cul9 might have a distinct substrate recruitment mechanism^29^ (Fig. 2).

## CRL ligases and adaptor proteins

Cullins, which have an evolutionarily conserved cullin homology domain, are essential proteins required for ubiquitin-dependent protein degradation. While all the cullin (and CRL) structures identified thus far are mammalian proteins, interactions between cullins and their CRL components have been analyzed using biochemical assays from yeast to humans, indicating conservation of the scaffolding functions of cullins. The core CRL comprises four components: a cullin protein that serves as a scaffold, a RING finger protein that binds to an E2 ubiquitin conjugating enzyme, a substrate receptor that recognizes the target protein, and adaptor proteins that connect the substrate recognition receptor to cullin. As different substrate receptors can target a variety of substrates, CRLs are involved in a wide range of biological processes and are associated with various diseases, including metabolic disorders. In this context, we will briefly discuss the common characteristics of adaptor proteins for CRLs.

### Skp1

CRL1, also known as SCF E3 ligase, is the most well-studied member of the CRL family. It consists of Cul1, Skp1, F-box protein, and Rbx1^30,31^. Cul1 acts as a scaffold that binds Skp1 and F-box proteins at its N-terminus and Rbx1 at its C-terminus. The F-box proteins determine the substrate specificity of the SCF complex. Rbx1 binds to E2 and facilitates ubiquitin transfer from E2 to substrates^32^. The activity of SCF E3 ubiquitin ligases is regulated by cullin neddylation, which disrupts inhibitory binding of cullin by CAND1 (Cullin Associated And Neddylation Dissociated 1)^33^. Cul8, similar to Cul1, acts as a scaffold that binds Skp1 at its N-terminus and Fbxw8 at its C-terminus. However, unlike SCF, Fbxw9 is the only known substrate receptor for CRL7.

### EloBC

CRL2 and CRL5 are two members of the CRL family that are structurally similar to CRL1. However, unlike CRL1, they utilize the obligate heterodimer of Elongin B and Elongin C (EloBC) as their adaptor component instead of Skp1. Cul2 and Cul5, the scaffold proteins for CRL2 and CRL5, respectively, recruit different substrate receptors. VHL box substrate receptors interact with Cul2/EloBC, while SOCS box proteins bind Cul5/EloBC. The VHL box motif and SOCS box motif are similar in both sequence and structure, which makes it difficult to predict their assembly with either Cul2 or Cul5^34^. Both motifs consist of a BC box and a cullin box. The BC box mediates the association with EloBC, while the cullin box is essential for Cul2/Cul5 specificity.

### BTB proteins

In many CRL complexes, the substrate recognition modules are constructed by isolated linker and adaptor proteins. However, Cul3 is a critical scaffold protein for ubiquitination, and a single BTB-containing adaptor can bind Cul3 scaffolded to substrates using two protein interaction sites, forming up to 188 Cul3-BTB E3 ubiquitin ligase complexes in mammals^35^. The BTB domain, structurally homologous to the adaptors Skp1 and EloC in CRL1 and CRL2/5, respectively, is involved in protein homo and heterodimerization, as well as multimerization.

BTB proteins can dimerize to control substrate ubiquitination. This leads to an assembled CRL3 that has two substrate receptors and two catalytic RING domains, which can either act independently to target two substrates or work together to target a single protein. The BTB protein KEAP1 recognizes two distinct degrons present in the same target substrate, NRF2^36^. Multimeric CRL3 assemblies can also be formed through BTB proteins containing BACK domains, such as SPOP, resulting in increases in E3 ubiquitin ligase activity or the concentration of CRLS^37^. Many BTB proteins, which contain unique DNA-binding zinc fingers (ZFs), also serve as transcriptional regulators^38^.

### DDB1

Cul4-containing CRLs are unique compared to other CRLs, as they utilize a different adaptor protein called DDB1, which lacks the BTB fold found in CRL1/2/3/5 adaptors. Instead, DDB1 is composed of three WD40 β-propeller domains (BPA, BPC, BPC) and a helical C-terminal domain. The BPB domain of DDB1 interacts with Cul4 and is structurally different from that of other adaptor proteins^39^. Cul4-DDBs recognize noncanonical substrates, such as damaged DNA. For instance, CRL4-DDB2 detects UV-induced lesions in DNA and ubiquitinates nearby proteins to facilitate nucleotide excision repair^40^. Additionally, small molecules can modulate the substrate specificity of a CRL.

## Roles of ring-finger E3 ligases and their adaptors in metabolic regulation

Ubiquitination plays a critical role in maintaining homeostasis and responding to various stress stimuli. Dysregulation of the ubiquitination process can lead to various human diseases, including metabolic disorders. E3 ubiquitin ligases are essential for modulating cellular homeostasis due to their regulation and substrate specificity during the ubiquitination cascade. RING-type E3 ligases are a large family of enzymes that regulate the stability and activity of numerous interaction substrates. Significant progress has been made in understanding the potential roles of RING-type E3 ligases in human health^39^. However, despite our increased awareness of this process, mechanistic links between E3 ubiquitin ligases, their adaptors, and substrates in human metabolic diseases need further elucidation. In this context, specific RING-type E3 ubiquitin ligases and their adaptors that are involved in metabolic diseases can be targeted for therapeutic interventions.

### The TRIM superfamily in diabetes mellitus and its complications

The tripartite motif-containing (TRIM) protein family is a highly conserved group of E3 ligases with 77 members identified in humans. Most of these proteins contain a RING-finger domain, one or two B-box domains, and a coiled-coil domain^41^. Dysregulation of TRIM proteins has been found to play crucial roles in multiple biological processes, including antiviral defense, regulation of immune and cellular stress responses, cell proliferation, apoptosis, cell differentiation, and DNA repair^42^.

TRIM proteins, acting directly or indirectly as regulatory proteins, play a role in the development of diabetes mellitus (DM) and its complications. For instance, TRIM27 deficiency led to increased T-cell infiltration, caspase-3 cleavage signal, and decreased β-cell mass, predisposing mice to streptozotocin-induced DM^43^. TRIM32 was found to mediate insulin resistance in the liver and skeletal muscle tissue by inhibiting plakoglobin function, which is involved in muscle atrophy^44^. However, TRIM72 (also known as MG53) degrades insulin receptor and insulin receptor substrate 1 (IRS1), thereby inhibiting the insulin signaling pathway^45,46^, and the upregulation of myocardial TRIM72 induces diabetic cardiomyopathy^47^. TRIM63 (muscle RING finger-1, MuRF-1) was identified as a ubiquitin ligase that is upregulated in various muscle atrophy conditions^48^, including diabetes-related muscle atrophy^49^. Therefore, pharmacologic agents that regulate the interaction between TRIMs and their substrates are considered potential therapeutic candidates for the treatment of metabolic diseases. For example, MID-00935, an inhibitor of the binding of TRIM72 to IRS1, was identified as a potential drug candidate for treating insulin resistance^50^. A recent study showed that MyoMed-205 treatment blunted the upregulation of TRIM72 and TRIM63 in obese muscle, resulting in improved myocardial diastolic function and prevented muscle atrophy^51^. In summary, several TRIM family proteins are closely associated with the pathogenesis of multiple metabolic complications.

### The TRAF family in glucose metabolism

The tumor necrosis factor receptor-associated factor (TRAF) family is characterized by two domains, the TRAF-N-domain and TRAF-C domain. Most TRAFs possess a RING domain at the N-terminus, and intracellular signaling molecules bind to the TRAF-N domain^52,53^. This family acts as adaptor proteins that mediate cytokine signaling and overtly regulate cell proliferation and the stress response^54^. There are six classic members (TRAF1-TRAF6) and one nonclassic member (TRAF7) known in mammals^55^. The structural features of TRAFs allow them to function as cytoplasmic junctions that promote intracellular signaling by binding to receptors and enhancing the recruitment of proteins to signaling complexes^56^.

TRAF4 and TRAF6 control the PI3K/AKT pathway by promoting AKT membrane recruitment and phosphorylation by serine/threonine kinases^57^. Previous studies revealed that treatment with 6877002, a small-molecule inhibitor that blocks the CD40-TRAF6 interaction, improved insulin sensitivity, reduced adipose tissue inflammation, and decreased hepatic steatosis^51,58^. Additionally, the hepatocyte-specific deletion of TRAF2 reduces the hyperglycemic response to glucagon and protects against hyperglycemia and hyperinsulinemia in obesity. However, it does not alter insulin signaling in the liver. This suggests that TRAF2 may act downstream of inflammation and/or ER stress to promote the hyperglycemic response to counterregulatory hormones^59^.

### The MARCH family in glucose and lipid metabolism

Membrane-associated RING-CH-type finger (MARCH) proteins are a subfamily of RING-type E3 ubiquitin ligases^60^. There are over 10 mammalian members of the MARCH family, all of which have E3 ubiquitin ligase activity and RING-CH domains^61^. MARCH6, also known as TEB4 and RNF176, is a member of this protein family and contains transmembrane domains and a RING-CH domain^62^. MARCH6 is responsible for targeting at least 10 different substrates, including itself, for rapid proteasomal degradation. Its functions have been largely associated with lipid metabolism. It is stabilized by cholesterol and controls the levels of lipid-related substrates, including four cholesterol synthesis enzymes^63^.

MARCH6 is a crucial protein involved in regulating sterol metabolism through the degradation of multiple enzymes within the cholesterol synthesis pathway. Specifically, MARCH6 targets 3-hydroxy-3-methyl-glutaryl coenzyme A reductase (HMGCR) and squalene monooxygenase (SM), two rate-limiting enzymes in cholesterol synthesis^64,65^. In addition, two enzymes involved in cholesterol biosynthesis, lanosterol 14α-demethylase (LDM/CYP51A1) and 24-dehydrocholesterol reductase (DHCR24), have been identified as new substrates of MARCH6^66^. Therefore, MARCH6 rapidly and effectively shuts down cholesterol production at multiple points within the pathway. MARCH6 targets several other lipid-related substrates, including perilipin-2 (PLIN2), Niemann-Pick disease (NPC) intracellular cholesterol transporter 1 (NPC1), and the bile salt export pump (BSEP). PLIN2 is involved in the formation of lipid droplets, and its interaction with MARCH6 may impact lipid droplet formation^67^. NPC1 is involved in cholesterol trafficking, and MARCH6 targets a mutant form of NPC1 as part of the quality control process in the ER^68^. The B238V mutant of BSEP, another MARCH6 substrate, is likely misfolded and mislocalized to the ER, with only some correctly trafficked to the plasma membrane^69^.

Few studies have revealed the involvement of other MARCH members in metabolism. For example, MARCH1 can regulate insulin receptor signaling by targeting it for ubiquitin-dependent downregulation^70^. Thus, MARCH1 deficiency in liver and adipose tissue leads to improved glucose clearance, and MARCH1 overexpression impairs clearance^70^. Additionally, a more recent study demonstrated that MARCH1 knockout (KO) mice show improved glucose handling, while female MARCH KO mice exhibit excessive weight gain and visceral adiposity^71^. These findings suggest that MARCH1 KO mice may be useful in identifying sex-specific metabolic regulation. Collectively, MARCH family members appear to play a central role in regulating sterol and lipid metabolism, although many avenues of investigation into this E3 ubiquitin ligase remain to be explored.

### The KLHL family in metabolism

The Kelch-like (KLHL) gene family encodes proteins that typically have a BTB/POZ domain, a BACK domain, and five to six Kelch motifs, and many of them interact with Cul3 to mediate ubiquitination^72,73^. KLHL3 is known to bind to with-no-lysine-kinases (WNKs), leading to their degradation^74^. Mutations in *WNK1*, *WNK4*, *CUL3*, and/or *KLHL3* in humans result in Gordon’s syndrome, a form of hypertension that is heritable and associated with salt-sensitive hypertension, hyperkalemia, metabolic acidosis, and thiazide sensitivity^75^. In mice, deletion of WNK4 reduces high-fat diet (HFD)-induced obesity^76^. KLHL3 also plays different roles in type 1 diabetes (T1D) and type 2 diabetes (T2D), with hyperglycemia causing increased NEDD8 in diabetic mice, resulting in increased degradation of KLHL3^77^. However, hyperinsulinemia indirectly affects KLHL3 phosphorylation. Dysregulation of KLHL3 may be related to electrolyte disturbances and hypertension in patients with diabetes^77^. Recent studies have shown that KLHL3 deficiency in mice prevents diet- and age-induced obesity and mitigates insulin resistance and NAFLD^78^. These findings suggest that KLHL3 may be a valuable therapeutic target for obesity and obesity-related metabolic diseases. However, further experiments are needed to clarify the relevant pathogenesis due to the limited number of studies available.

The inducible transcription factor nuclear factor erythroid 2-related factor 2 (NRF2) regulates the expression of several hundred genes encoding proteins with various homeostatic functions, including antioxidant, anti-inflammatory, and drug metabolism functions. The activity of NRF2 is mainly regulated by the redox sensor protein Kelch-like ECH-associated protein 1 (KEAP1, also known as KLHL19), which functions as an E3 ubiquitin ligase adaptor for proteasomal degradation of NRF2. KEAP1 also serves as a sensor for endogenous and exogenous electrophiles and oxidants, as it contains several highly reactive cysteine residues that prevent its ability to target NRF2 for degradation. This results in the accumulation of NRF2 and the upregulation of a large network of cytoprotective proteins^79^. In addition to NRF2, KEAP1 has multiple binding partners, which in turn have roles in various cellular processes.

Although NRF2 is often hyperactive in established human tumors, where it contributes to the hallmarks of cancer, its contributing role to susceptibility to nonneoplastic diseases is also well documented^80^. KEAP1-knockdown mice have been shown to exhibit enhanced NRF2 signaling in the liver, which attenuated methionine- and choline-deficient (MCD) diet-induced fatty liver by increasing hepatic antioxidant and detoxification capacity^81^. A global analysis of mouse hepatic gene expression identified that both genetic and pharmacologic activation of NRF2 induced a large number of genes associated with lipid and glucose metabolism, rather than xenobiotic detoxification. Additionally, this activation tended to reduce lipid synthesis in the liver, supporting the idea that the NRF2/KEAP1 axis suppresses lipid metabolism^82^. K67 is a specific inhibitor of the interaction between phosphorylated p62/Sqstm1 and KEAP1, and it suppresses the expression of NRF2 target genes^83^, making it a potential therapeutic candidate for the treatment of metabolic diseases.

### The KCTD family in glucose and lipid metabolism

The *potassium (K*^*+*^*) channel tetramerization domain* (KCTD) family consists of 25 partially characterized proteins, which are increasingly recognized for their roles in various biological functions^84^. These proteins share a conserved BTB/POZ domain^85^ that is essential for their diverse functions^86^. KCTD proteins can be classified based on their ability to bind E3 ubiquitin ligases through their BTB domain and participate in degradative processes. For instance, KCTD proteins from Group B (KCASH1^KCTD11^, KCASH2^KCTD21^, KCASH3^KCTD6^), Group C (KCTD10, -13, TNFAIP1), Group D (KCTD3, SHKBP1), Group E (KCTD2, -5, -9-, -17) and Group H (KCTD7, -14) can form an E3 ligase complex together with Cul3, selectively recruiting substrates for ubiquitination^87,88^. KCTD genes have been related to several diseases, including neurodevelopmental, neuropsychiatric, and neurodegenerative disorders^84,89^. Recently, several KCTD proteins have also been linked to cancer and metabolic diseases, although the mechanisms of their involvement are not yet fully understood.

Improper regulation of KCTD genes has been associated with obesity and altered concentrations of HDL cholesterol^90,91^. KCTD15 is a multifunctional protein involved in both neural crest formation and food uptake^92,93^. A recent study identified KCTD15 as a GRP78 partner throughout the adipogenesis process, suggesting that KCTD15 may be a new target for obesity control^94^. KCTD17 has known roles in ciliogenesis and synaptogenesis^95,96^, but its functions outside the central nervous system are not well known. Recent studies have explored its roles in the liver and adipose tissue. KCTD17 expression is increased in the livers of obese mice and patients with NAFLD/nonalcoholic steatohepatitis (NASH), leading to the degradation of pleckstrin homology domain leucine-rich repeat protein phosphatase 2 (PHLPP2) to prolong insulin signaling by dephosphorylating AKT^97^. Hepatocyte KCTD17 deletion in HFD-fed mice improved glucose intolerance and fatty liver by increasing carbohydrate response element-binding protein (ChREBP) protein stability via the degradation of O-GlcNAcase (OGA), suggesting that KCTD17 is a key regulatory node in multiple liver metabolic processes and a novel therapeutic option for the treatment of obesity-induced insulin resistance and NAFLD^98^. Additionally, its expression levels are increased in white adipose tissue in obese mice compared to lean control mice. KCTD17 binds to C/EBP homologous protein (Chop) to target it for ubiquitin-mediated degradation, which is likely associated with increased adipogenesis^99^, supporting KCTD17 as a novel therapeutic target for obesity. Validation of the interaction between KCTD family members and other substrates may identify novel roles of KCTD17 family members and open an interesting scenario for the design and characterization of new druggable targets in the regulation of metabolic diseases.

## Conclusion and future perspectives

**Fig. 3:**
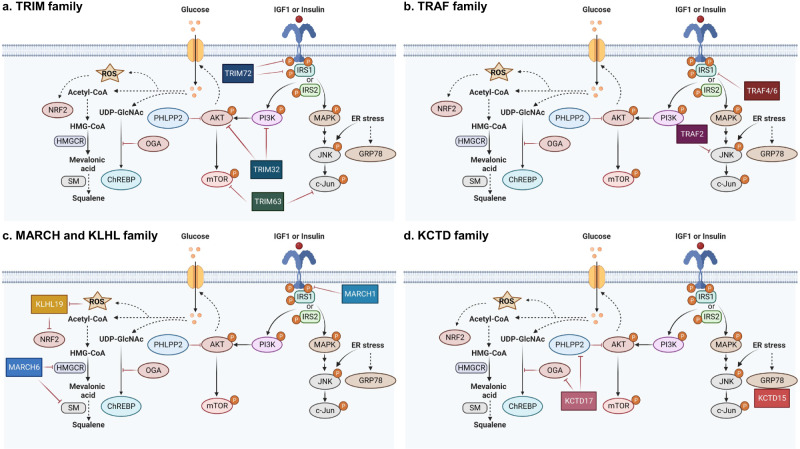
A pictorial representation of the relationship between E3 ubiquitin ligases and their adaptors in the metabolic pathway. **a**–**d** IRS insulin receptor substrate, PI3K phosphatidylinositol 3-kinase, PHLPP2 PH domain and leucine rich repeat protein phosphatase 2, mTOR mammalian target of rapamycin, MAPK mitogen-activated protein kinase, JNK c-Jun N-terminal kinase, OGA O-GlcNAcase, ChREBP carbohydrate response element binding protein, UDP-GlcNAc UDP-N-acetylglucosamine, HMG-CoA 3-hydroxy-3-methylglytaryl coenzyme A, HMGCR HMG-CoA reductase, SM squalene monooxygenase.

Metabolic diseases are a growing global health concern, and effective strategies are needed for prevention and treatment. One of the considerable strategies to control the occurrence or development of metabolic diseases is ubiquitin-mediated targeted protein regulation. In this review, we have briefly illustrated the principles of the ubiquitin system and the important role of E3 ligases and their adaptors in metabolic signaling (Fig. 3 and Table 1).

**Table 1:** Relationships between E3 ligase adaptors and metabolic regulation.

E3 ligase or adaptor	Substrates	Metabolic diseases or regulation	Small compound
TRIM Family			
TRIM27	RIP1	T1DM, β-cell mass	NA
TRIM32	PI3K, AKT	Insulin resistance, Diabetes-related muscle atrophy	NA
TRIM72 (MG53)	IRS1	Insulin signaling	MID-00935
TRIM63 (MuRF-1)	mTOR, c-Jun	Diabetes-related muscle atrophy	MyoMed-205
TRAF Family			
TRAF4, TRAF6	PI3K, AKT, IRS1	Insulin resistance, Glycolysis, Cell proliferation, Apoptosis	6877002
TRAF2	JNK	Obesity, ER stress, Hyperglycemia, Hyperinsulinemia	NA
MARCH Family			
MARCH6 (TEB4 or RNF176)	HMGCR, SM, LDM/CYP51A1, DHCR24, PLIN2, NPC1, BSEP	Cholesterol metabolism, Lipid metabolism	NA
MARCH1	IRS1	Glucose homeostasis	NA
KLHL Family			
KLHL3	WNK4, NEDD8	Obesity, NAFLD, T1DM, T2DM, Hyperglycemia, Insulin resistance	NA
KLHL19 (KEAP1)	NRF2	Oxidative stress, NAFLD, Lipid metabolism, Glucose metabolism, Cancer	K67
KCTD Family			
KCTD15	GRP78	Adipogenesis	NA
KCTD17	PHLPP2, OGA, CHOP	Obesity, NAFLD, NASH, Insulin resistance, Adipogenesis	NA

Targeted protein degradation through regulating the expression or activity of E3 ligases or their adaptors has gained much attention among the scientific community. However, a major gap in this area is the lack of understanding regarding the mechanisms that control E3 ubiquitin ligases or their adaptors. By developing therapeutic approaches that target specific E3 ubiquitin ligases or their adaptors, it is possible to stabilize target proteins without affecting the expression of others, resulting in more effective treatments for metabolic diseases with fewer adverse effects. Therefore, a better understanding of the complex biochemistry underlying the physiological functions regulated by E3 ubiquitin ligases and their adaptors is necessary to identify their potential roles as metabolic regulators and therapeutic targets.
